# Recent Advances in Manganese-Based Materials for Electrolytic Water Splitting

**DOI:** 10.3390/ijms24076861

**Published:** 2023-04-06

**Authors:** Jing Hu, Yuru Zhou, Yinan Liu, Zhichao Xu, Haijin Li

**Affiliations:** School of Energy and Environment, Anhui University of Technology, Ma’anshan 243002, China; zyr102181@163.com (Y.Z.); lyn100404@126.com (Y.L.); wenji7wu@163.com (Z.X.)

**Keywords:** manganese-based, hydrogen evolution reaction, oxygen evolution reaction, electrocatalysis, optimization strategy

## Abstract

Developing earth-abundant and highly effective electrocatalysts for electrocatalytic water splitting is a prerequisite for the upcoming hydrogen energy society. Recently, manganese-based materials have been one of the most promising candidates to replace noble metal catalysts due to their natural abundance, low cost, adjustable electronic properties, and excellent chemical stability. Although some achievements have been made in the past decades, their performance is still far lower than that of Pt. Therefore, further research is needed to improve the performance of manganese-based catalytic materials. In this review, we summarize the research progress on the application of manganese-based materials as catalysts for electrolytic water splitting. We first introduce the mechanism of electrocatalytic water decomposition using a manganese-based electrocatalyst. We then thoroughly discuss the optimization strategy used to enhance the catalytic activity of manganese-based electrocatalysts, including doping and defect engineering, interface engineering, and phase engineering. Finally, we present several future design opportunities for highly efficient manganese-based electrocatalysts.

## 1. Introduction

Energy is fundamental to human life. With the rapid development of the world economy and the rapid growth of population in the 21st century, human demand for energy is increasing day by day. According to identified reserves, the three traditional mineral resources, namely, coal, oil, and natural gas, are gradually becoming exhausted. Meanwhile, the issues of waste gas, the greenhouse effect, and ecological damage caused by the combustion of fossil fuels have become increasingly serious [1,2,3,4,5,6]. The recent outbreak of PM2.5 in China has alarmed the world. Developing clean and efficient renewable energy solves the problems of energy consumption and environmental pollution. In recent years, most countries have directed substantial technology and financial resources into the development and use of renewable energy for sustainable development [7,8,9,10,11]. Electrocatalytic water splitting for renewable electricity is widely regarded as a promising strategy for the large-scale production of high-purity hydrogen. Electrolytic water splitting consists of two half reactions: the hydrogen evolution reaction (HER) and the oxygen evolution reaction (OER) [11,12,13,14,15,16,17,18,19,20,21]. The efficiencies of the HER and the OER are severely limited by the inevitable dynamic overpotential [22,23]. The OER exhibits slow reaction kinetics and requires a high overpotential due to its multiple electron transfer process, which ultimately determines the overall efficiency of water splitting [24,25]. In view of this, efficient electrocatalysts are particularly important for the OER to reduce energy and improve energy conversion efficiency. To date, Ir/Ru-based noble metal materials are the benchmark for the OER. Electrolytic water splitting is a production process for high-purity hydrogen with great prospects [26,27,28].

The appearance of this noble metal catalyst reduces the overpotential of the OER and the HER, but its widespread use in industrial electrolytic cells is limited due to its high cost and scarcity. A large number of studies have focused on catalysts based on transition metals (mainly Mn, Fe, Co, and Ni), including nitrides, carbides, chalcogenides, and phosphates [29,30,31]. Among them, manganese-based materials have become the focus of many scientists. Manganese is an abundant, nontoxic, harmless transition metal with numerous valence states. Compared to traditional Fe-N-C catalysts, its larger electronic orbital configuration can significantly improve the degree of graphitization and oxidation resistance of the carbon support and eliminate the Fenton effect. Fenton oxidation is a chemical process that occurs under acidic conditions and involves the reaction between Fe^2+^ ions and hydrogen peroxide (H_2_O_2_), resulting in the formation of highly reactive hydroxyl radicals (OH) that possess strong oxidizing capacity. These hydroxyl radicals play a crucial role in initiating subsequent reactions, leading to the ultimate mineralization of organic molecules to CO_2_ and H_2_O [32,33,34,35,36]. Thus, the stability of the catalyst is improved effectively. Manganese-based materials can be prepared using various optimization strategies, resulting in a significant increase in specific surface area and abundance of active sites. As a result, these materials exhibit superior performance compared to commercial Pt/C catalysts [37,38,39]. Chen et al. synthesized an unusual α-MnO_2_ nanowire network through a mild hydrothermal reaction without any surfactants; the unique network structure of this nanowire resulted in improved hydrophilicity and conductivity, both of which are positive factors for efficient electrocatalysts [22]. Using a novel approach of creating heterogeneous structures between two different metal sulfides, Zhang et al. synthesized a highly efficient material, S-doped carbon bridged semi-crystalline MIL-based Co_3_S_4_/MnS_2_, demonstrating the potential of MOFs-assisted structural engineering to improve catalytic performance in transition metal sulfides heterostructure electrocatalysts [40]. Wang et al. synthesized Mn_2_P-Mn_2_O_3_ heterogeneous nanoparticles through a one-pot method. The heterogeneous structure provided abundant active sites for HER and OER, optimized the electronic structure, and promoted the adsorption of H* [41].

**Figure 1:**
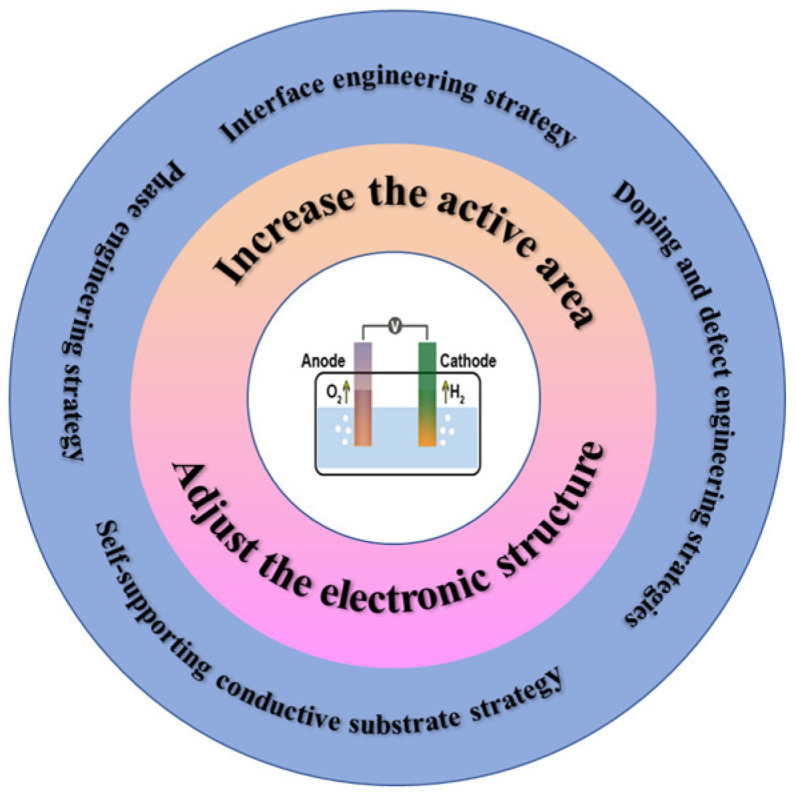
Schematic diagram of the engineering strategy for manganese-based materials.

In this review, we focus on the latest developments and breakthroughs in the regulation strategies of manganese-based electrocatalysts. We first introduce the mechanism of the electrocatalytic decomposition of water by manganese-based electrocatalysts. We then focus on the general strategies and recent advances in regulating the performance of manganese disulfide electrocatalysts, including doping and defect engineering, interface engineering, self-supported conductive substrates, and phase engineering strategies. We also discuss the regulation mechanism of each strategy. Finally, we summarize the current unsolved challenges in enhancing the electrocatalytic activity of manganese-based electrocatalysts for electrolytic water splitting and propose possible solutions (Figure 1).

## 2. Basic Knowledge of Manganese-Based Materials

This work mainly introduces the type, crystal structure, electronic structure, and mechanism of electrocatalytic decomposition of water by manganese-based electrocatalysts to provide the readers with a preliminary understanding of the material family. 

At present, manganese-based materials mainly include highly active and low-cost catalysts, such as manganese oxides (Mn_2_O_3_, Mn_3_O_4_ and MnO_2_), manganese sulfides (MnS, MnS_2_), and manganese selenide (MnSe). The high activity of manganese oxide catalysts in the catalytic oxidation of volatile organic compounds (VOCs) is primarily influenced by factors such as their morphology, specific surface area, oxygen vacancy, and surface composition. MnO_2_ has been received special attention because of its good catalytic activity and low cost. MnO_2_ is a crystal or black powder with up to 30 different crystal structures. As a natural mineral, it is more complex than simple oxides due to its polyvalence and nonstoichiometric composition [42,43,44,45,46]. In catalysis, different crystalline MnO_2_ types have different catalytic activities. In addition, for MnO_2_ of the same crystal type, different structures often have different catalytic properties, such as 1D nanostructures, hollow structures, lamellar structures, and rod-like linear structures. Owing to the polycrystalline structure and fully exposed active sites of Mn, this structure has excellent electrocatalytic activity for the oxygen evolution reaction during water electrolysis. One of the remarkable characteristics of MnO_x_ materials is the variety of crystal structures. MnO_2_ is piled up to form various crystalline phases by edge sharing and corner sharing of basic structural units (namely, the MnO_6_ octahedron, which has numerous morphologies and structures and is prone to phase transformation). The common MnO_2_ crystal structure consists of α, β, γ, δ, and λ phases, and its size depends on the number of connected [MnO_6_] octahedral elements (n × m) [47,48,49,50,51]. The tunnel structure and layer structure formed by different connection modes often contain a variety of metal cations. The connection modes of a wide variety of MnO_6_ octahedrons form a (1 × 1) pyrolusite (β-MnO_2_) and cryptocrystalline (2 × 2) tunnel structure (α-MnO_2_). Large tunnels facilitate ion diffusion, so the capacity of γ-MnO_2_ and α-MnO_2_ in nanostructures is higher than that of β-MnO_2_. Cheng et al. studied the effect of the crystal phase structure on the catalytic activity of MnO_2_ and found that the catalytic performance of their efficient oxygen reduction reaction (ORR) follows the order of β-MnO_2_ < λ-MnO_2_ < γ-MnO_2_ < α-MnO_2_ [52]. Xue et al. successfully synthesized MnO_2_ with different crystal shapes and morphologies in the system of manganese chloride–potassium permanganate by a controllable redox reaction using a microwave hydrothermal method [53].

**Figure 2:**
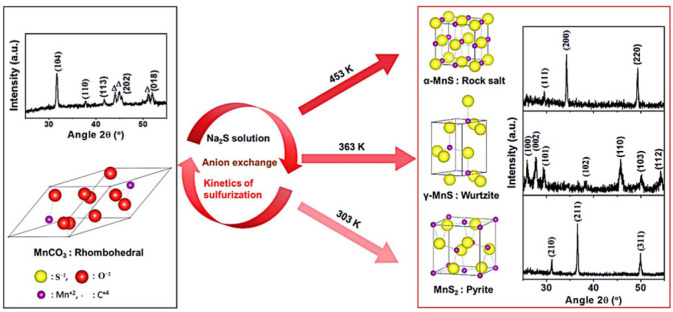
Structure of MnS at different temperatures. Reproduced with permission from Ref. [60]. Copyright 2020, The Royal Society of Chemistry.

Transition metal dichalcogenides (TMDs), usually labeled as MX_2_ (M: transition metal elements from groups IVB to VIII; X: S, Se, and Te elements of group VIA), are typical 2D layered nanomaterials. Each TMD monolayer contains three atomic layers, forming an “X-M-X” sandwich structure. Adjacent monolayers are 6–7 Å apart and held together by weak van der Waals forces, which makes them easy to peel away [54,55,56]. Manganese sulfide, MnS, is a weakly magnetic semiconductor material of groups VIIB–VIA, with a band gap width of 3.7 eV. Manganese sulfide has four different crystal structures: the cubic structure, MnS_2_; the green, stable halite structure, α-MnS; the pink, metastable structure, β-MnS; and the pink, metastable fiber catalpa frame structure, γ-MnS. Metastable β- and γ-MnS are easily transformed into α-MnS at 100–400 °C or at high pressure [57,58,59]. Compared with that at the stable state, the metastable manganese sulfide shows more special chemical and electrical properties. In recent years, the preparation methods of manganese sulfide include electrodeposition, electrospinning, the chemical vapor phase method, the sol–gel method, and the hydrothermal method. Pujari et al. prepared MnS with different crystalline phases by controlling the temperature. MnS_2_ was obtained when the hydrothermal temperature was 300 K, γ-MnS was obtained at 363 K, and α-MnS was generated at 453 K. The study also found that different reaction methods, reactants, times, or solvents had a great influence on the morphology of manganese sulfide (Figure 2) [60]. Manganese selenide (MnSe) is a non-layered material with a wide band gap of about 2.0 eV which belongs to the TMDC family. It has photoelectric, magnetic, and a variety of crystal structures, and exhibits a stable structure and high conductivity. Due to its high magneto-optical properties and conductivity, it has been widely used as a cathode material for batteries as well as photoelectrochemical water splitting. This paper mainly studies the optimization strategy of manganese oxides and manganese sulfide in the field of electrocatalytic water splitting, and the photoelectric properties of manganese selenide and other manganese-based materials need to be further explored in the use of electrocatalytic water splitting, which presents an interesting research direction [61].

The mechanism of the electrocatalytic decomposition of water by manganese-based electrocatalysts involves the transfer of electrons to the catalyst surface, which enables the catalyst to lower the energy barrier for water oxidation and/or reduction. Specifically, manganese-based catalysts can act as efficient water oxidation catalysts by facilitating the formation of oxygen from water molecules, while also promoting the release of protons and electrons. This mechanism involves a series of intermediate steps, including the formation of reactive oxygen species, the transfer of protons and electrons, and the release of oxygen gas. Overall, the electrocatalytic decomposition of water by manganese-based electrocatalysts is a complex process that involves multiple chemical and electrochemical reactions, which can be influenced by a range of factors, including the structure, composition, and morphology of the catalyst material.

The hydrogen evolution reaction and oxygen evolution reaction exhibit different behaviors in different electrolytes, and the reaction process is also different. The HER, regardless of the medium the reaction is in, involves the adsorption and desorption of H^*^, and the reaction rate is determined by the adsorption capacity of the electrocatalyst to H. The reaction process of the OER is more complex than the HER, involving the transfer of multiple electrons and the formation of O-O bonds, and only the O-H bonds need to be broken during the reaction due to the presence of OH^−^ in basic and neutral solutions. In the oxide electrocatalyst of manganese, Mn^3+^ is the key to electrocatalysis. Mn^3+^ has a long Mn-O-Mn bond, so Mn-O has high flexibility, favoring the formation of Mn-OH_2_ and the fracture of Mn-O_2_, which is conducive to the progress of OER. Metal sulfides or selenides can be converted into corresponding metal oxides/hydroxides, and the converted substances are considered to be important active sites for sulfide/selenide catalysts. In addition, surface residues or Se in oxides/hydroxides will further reduce the adsorption-free energy difference between O^*^ and OH^*^, which is important reason the high electrocatalytic activity [62,63].

## 3. General Strategies and Progress in Regulating the Performance of Manganese-Based Electrocatalysts

### 3.1. Doping and Defect Engineering Strategies

**Figure 3:**
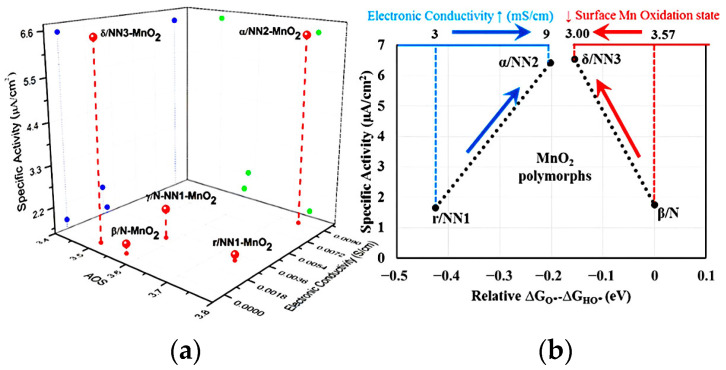
(**a**) Specific OER activities of different polymorphs MnO_2_ [β/N-, γ/N-NN_1_-, r/NN_1_-, α/NN_2_-, and δ/NN_3_-MnO_2_] with their oxidation state of Mn (activated oxygen species (AOS)) and bulk electronic conductivities. Blue and green dots represent projection in specific activity–electronic conductivity and specific activity–AOS planes, respectively. (**b**) Volcano-based relationship for the specific OER activity of MnO_2_ polymorphs with the computed universal descriptor ΔG_O*_−ΔG_HO*_ (eV) (relative to the native phase β/N-MnO_2_). Reproduced with permission from Ref. [69]. Copyright 2019, American Chemical Society.

Defect engineering is regarded as an effective method to improve the electronic structure and physicochemical properties of electrode materials and has been widely used in recent years. Point defects are the main topic of defect chemistry research, which is mainly divided into two categories: intrinsic defects and non-intrinsic defects. The formation of defects does not change the composition of the entire crystal. Intrinsic defects, which are caused by the thermal vibration of lattice atoms, are an integral part of the intrinsic crystal and include Schottky defects and Frenkel defects. Non-intrinsic defects are caused by impurity atoms or impurity ions embedded in the lattice, so they are also called doping defects. Introducing a vacancy (such as an O, S, or N vacancy) into electrocatalysts is a valuable defect engineering strategy to improve the behavior of electrocatalysis [64,65,66,67]. For example, Zhao’s research group used a new type of dual-function electrode material composed of two single-layer-thick δ-MnO_2_ nanosheet arrays and found that the nanomaterial dual-function electrode exposed many active sites of the electrocatalytic reaction. They performed density functional theory (DFT) calculations to determine the main factor affecting the catalytic performance and found that the ultrathin δ-MnO_2_ nanosheets contain abundant oxygen vacancies, leading to the formation of Mn^3+^ active sites. As a result, electrical conductivity is enhanced [68]. Gupta et al. achieved improved OER performance by stabilizing the metastable or unnatural forms of electrocatalysts and revealed correlations between OER and variable oxidation states and electron conductivity, thus providing directions for generalizing these effects to other polymorphic compounds. α/NN_2_-MnO_2_ has a high electrical conductivity and specific activity, which are related to its low oxygen vacancy formation energy. Based on the general descriptor ΔG_O*_−ΔG_HO*_ calculated by DFT, a volcano-based relationship for the specific OER activity of MnO_2_ morphology was observed. The X-ray photoelectron spectroscopy (XPS) representation and DFT calculation indicate that the formation of low oxygen vacancy, the high specific activity of δ/NN_3_-MnO_2_, and the electronic origin of the high OER activity are attributed to the Mn-d valence band moving closer to the Fermi level, leading to strong O adsorption (Figure 3) [69].

**Figure 4:**
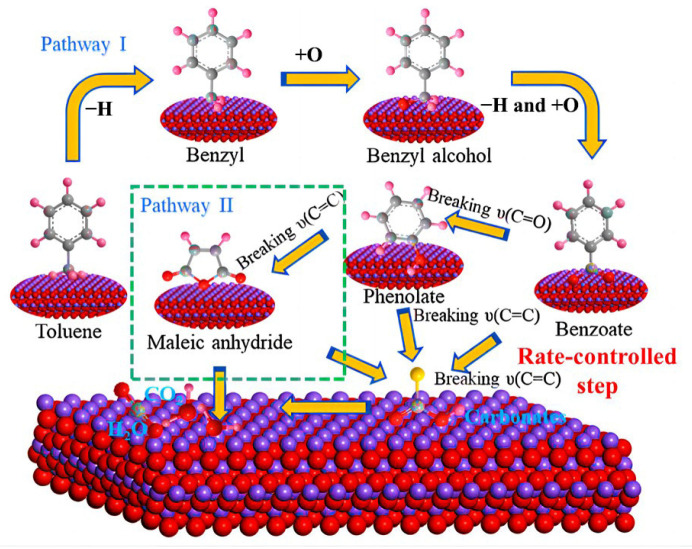
Proposed mechanism for toluene oxidation on a manganese-based catalyst. Reproduced with permission from Ref. [70]. Copyright 2020, Elsevier B.V.

Mo et al. used various manganese-based oxides to oxidize toluene by adjusting the solvent and double complexation pathway. It was concluded that the oxygen vacancy of the catalyst increased and thus accelerated the oxidation of toluene. They also found that the toluene on the surface of the manganese-based catalyst can be oxidized into various intermediates, the oxygen species and lattice oxygen adsorbed on the surface of the catalyst can participate in the partial activation of toluene molecules at the same time, and the surface adsorbed oxygen species of gas-phase oxygen supplemented by oxygen vacancies can effectively react with the adsorbed toluene molecules to form H_2_O and CO_2_. The two main pathways were pathway I (toluene-benzyl-benzyl alcohol-benzoate-short-chain carbonate H_2_O and CO_2_ pathway) and pathway II (toluene-benzyl-benzyl alcohol-benzoate-phenolate-maleic anhydrides-short-chain carbonates-H_2_O and CO_2_). The rapid C=C cleavage of benzoate may be the rate-determining step of the manganese-based catalyst during the entire oxidation. The resulting oxygen vacancy is likely to adsorb and activate the gaseous oxygen supplement in the catalyst surface and improve the oxygen migration rate and oxygen storage capacity. XPS further revealed that the enrichment of adsorbed oxygen as active oxygen and the increase in Mn^4+^ concentration could improve the oxidation activity of toluene (Figure 4) [70].

**Figure 5:**
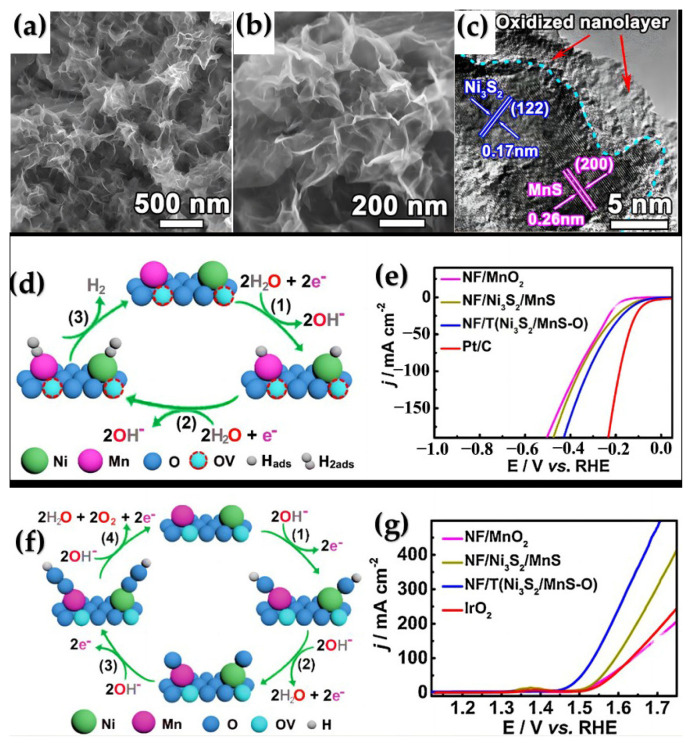
(**a**,**b**) Scanning electron microscopy (SEM) image and (**c**) High-resolution transmission electron microscopy (HRTEM) image of NF/Ni_3_S_2_/MnS nanosheets. (**d**) Schematics of the HER pathways with Mn and Ni atoms adjacent to OV as active sites. Only one OV near the Mn or Ni atom is illustrated (**e**) HER polarization curve with a scan rate of 10 mV s^−1^ at NF/T(Ni_3_S_2_/MnS-O), NF/Ni_3_S_2_/MnS, NF/MnO_2_, and the commercial Pt/C in 1.0 M KOH. (**f**) Schematics of the OER pathways with Mn and Ni atoms adjacent to OV as active sites. Only one OV near Mn or Ni atom is illustrated. (**g**) OER polarization curve with a scan rate at 10 mV s^−1^ of NF/T(Ni_3_S_2_/MnS-O), NF/Ni_3_S_2_/MnS, NF/MnO_2_, and commercial IrO_2_ in 1.0 M KOH. Reproduced with permission from Ref. [75]. Copyright 2019, Elsevier B.V.

Furthermore, through a hydrothermal reaction, Zhang et al. synthesized Ni_3_S_2_/MnS on nickel foam (NF), which exhibited slight electro-oxidation to produce NF/T (Ni_3_S_2_/MnS-O) with an abundance of oxygen vacancies. This material can serve as a bifunctional electrode for overall water splitting, thanks to its layered porous structure and high active surface area, with fully exposed oxygen vacancies (OVs). Compared to MnO_x_ and other metal oxides/sulfides, Ni_3_S_2_/MnS has superior dual-function performance in the HER and the OER, including a highly efficient electrocatalytic activity and greater stability (Figure 5a–c) [68,71,72,73,74]. The Ovs adjacent to M (M=Mn or Ni atom) sites lower the free energy required for the adsorption process of H_ads_ on M, which forms M-H_ads_ (Figure 5d, Step 1), as well as for the desorption of H_2_ (Steps 2–3). Additionally, the adsorption of OH^−^ promotes the formation of M-OH_ads_ (Figure 5f, Step 1). In the subsequent rate-determining step, Ovs decrease the free energy needed for the conversion of M-OH_ads_ to active intermediates (MO_ads_, M-OOH_ads_, Steps 2–3). The overvoltage was 0.31 V at a current density of 10 mA cm^−2^, which is lower than that of Pt/C//IrO_2_ (1.56 V, 0.33 V) (Figure 5e,g). This finding indicates that metal oxide layers rich in Ovs are beneficial to improve electrocatalytic performance. Therefore, oxygen vacancies can be induced by electrooxidation to obtain high-efficiency nonprecious metal electrocatalysts [75].

**Figure 6:**
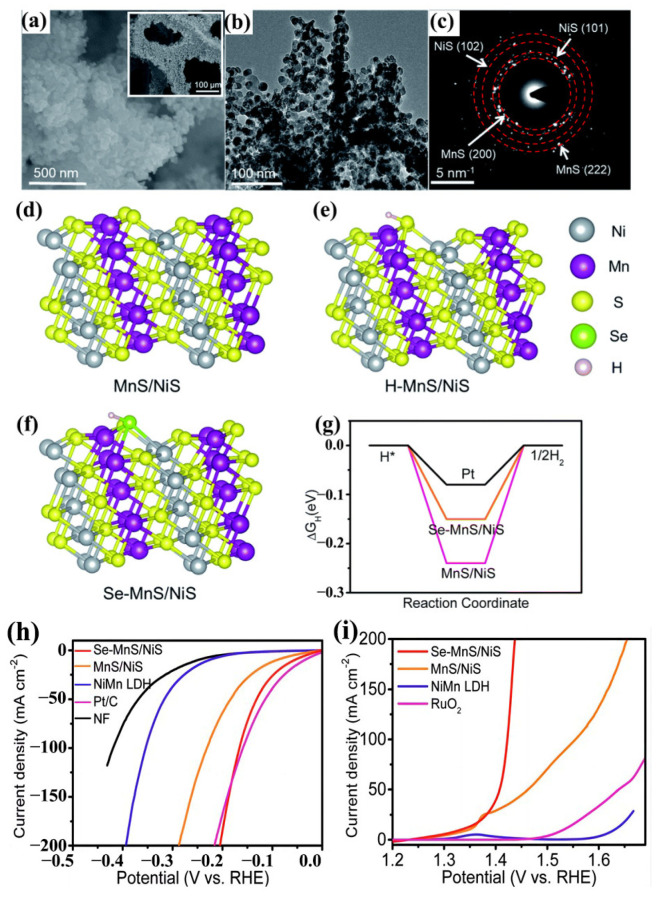
(**a**) SEM and (**b**) TEM images of Se-MnS/NiS. (**c**) Selected area electron diffraction (SAED) pattern of Se-MnS/NiS. (**d**–**f**) Structures. (**g**) Free energy diagram of HER over MnS/NiS and Se-MnS/NiS. (**h**) HER polarization curves of NiMn LDH, MnS/NiS, Se-MnS/NiS, NF, and Pt/C in 1 M KOH at 10 mV s^−1^. (**i**) OER polarization curves of Ni-Mn LDH, MnS/NiS, and Se-MnS/NiS in 1 M KOH with a scan rate of 10 mV s^−1^. Reproduced with permission from Ref. [76]. Copyright 2019, The Royal Society of Chemistry.

In summary, defect engineering is a widely used method in experiments where vacancies can be used as the active site of water adsorption/desorption, reduce the energy barrier, generate optimal reaction kinetics, and provide a way to solve the problems existing in manganese-based materials. The introduction of heteroatoms (doping engineering) into materials can bring various advantages for electrocatalytic applications. Theoretical calculations indicate that the introduction of heteroatoms can create defects in the structure of 2D TMDs, leading to an adjustment in their band gap and redox capacity. Zhu et al. synthesized Se-MnS/NiS electrocatalysts by a hydrothermal reaction and chemical deposition for the first time. The introduction of selenium dopants can adjust the structure and increase the electrochemically active surface area, and the conductivity is improved due to the formation of Ni-S and Mn-S bonds (Figure 6a–c). The synergistic effect of Se-MnS/NiS heterojunction promotes the adsorption of hydrogen atoms on the catalyst surface, which is conducive to the electrocatalysis of the HER and the OER. Meanwhile, the effect of selenium doping on the HER catalytic activity of MnS/NiS was further studied by DFT calculations. The adsorption free energy (ΔG_H*_) of hydrogen in Se-MnS/NiS was 0.15 eV, which is lower than that of MnS/NiS (0.24 eV), indicating that the HER of MnS/NiS after Se-doping has ideal H^*^ adsorption kinetics (Figure 6d–g). Through the electrocatalytic test of HER and OER, it is known that its overpotential is better than that of the original material at a constant current density (Figure 6h,i) [76].

Zhao et al. doped Fe into Ni_3_S_2_/MnS. The improved catalytic activity is attributed to the combination of heterojunction that improves the conductivity of the catalyst, adjusts the electronic configuration of the active site, and optimizes the adsorption capacity of oxygen-containing intermediates to achieve rapid reaction kinetics and high catalytic performance [77]. Doping metal into the original catalyst can improve the catalytic performance and change the electronic structure, which is also a widely used method. Gupta et al. directly doped manganese into nanocarbon composites and found that catalytic activity and stability of carbon nanostructure (Fe-Co-Ni) could be improved. In addition, β-MnO_2_ could be generated on the surface of the carbon structure. The substance not only improves the catalytic performance but also adheres to the surface to significantly reduce carbon corrosion, protecting the active sites [78]. These experiments demonstrate that the proper integration of transition metal doping engineering can increase the active sites and enhance the stability, a common strategy in experiments.

### 3.2. Interface Engineering Strategy

**Figure 7:**
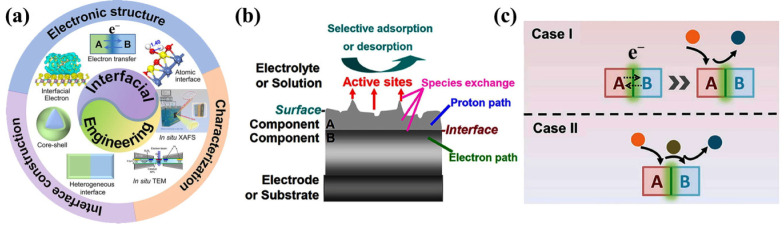
(**a**) Illustration of interfacial engineering of heterogeneous catalysts. Reproduced with permission from Ref. [85]. Copyright 2021, Elsevier B. V. (**b**) Illustration for an ideal simple triple-phase boundary structure where the electron path, proton path, and solution phases are presented together with active sites. Reproduced with permission from Ref. [84]. Copyright 2015, Elsevier B. V. (**c**) A catalyst composed of component A and component B with an interface between both of them. In case I, A provides the active sites for the catalytic reaction. B modifies the surface environment of A for optimal electrocatalysis. In case II, the adsorption and desorption processes occur on A and B separately. Reproduced with permission from Ref. [83]. Copyright 2018, Wiley-VCH.

Constructing heterogeneous catalysts with high active interfaces, especially metal cluster catalysts, is an effective strategy to improve metal catalytic activity, stability, and atomic utilization. The strong interaction, coordination effect, synergistic effect, and limited domain effect at the interface have a great influence on the activity and stability of the catalyst. The interface structure is usually formed between two or more different components and can theoretically be used as a transport channel for electrons or intermediates between different components [79,80,81]. The physical and chemical properties of electrocatalysts can be regulated by reasonably controlling the interfacial atomic arrangement, which has a great impact on the electrocatalytic performance. We summarize the current research progress on the interfacial engineering of heterogeneous catalysts. Pan et al. prepared Pt-free 1T/2H-MoS_2_/CdS/MnO_x_ hollow-pore nanocomposites by a hydrothermal method. Electrocatalytic reactions generally occur on the surface or interface of the catalyst, where multielectron transfer occurs and reaction intermediates can be found [82]. Owing to the synergistic effect between components, two or more materials participate in the reaction, electrons are redistributed at the interface, and multiple forms of interfaces are established in the materials and intermediates (Figure 7a). The author introduced the transfer mode of electrons and protons between different material interfaces and analyzed the reaction mechanism between materials. In case 1, a bridge was built between A and B for electron transfer, with A as the main part of the catalytic reaction. In case 2, a direct co-reaction occurred from A to B, where electron transfer transpires at the interface between A and B (Figure 7b,c) [83,84,85]. The different components of the heterogeneous electrocatalyst have different effects on the catalytic reaction. The catalytic activity of the heterogeneous electrocatalyst is better than that of each component; this phenomenon is known as synergy.

**Figure 8:**
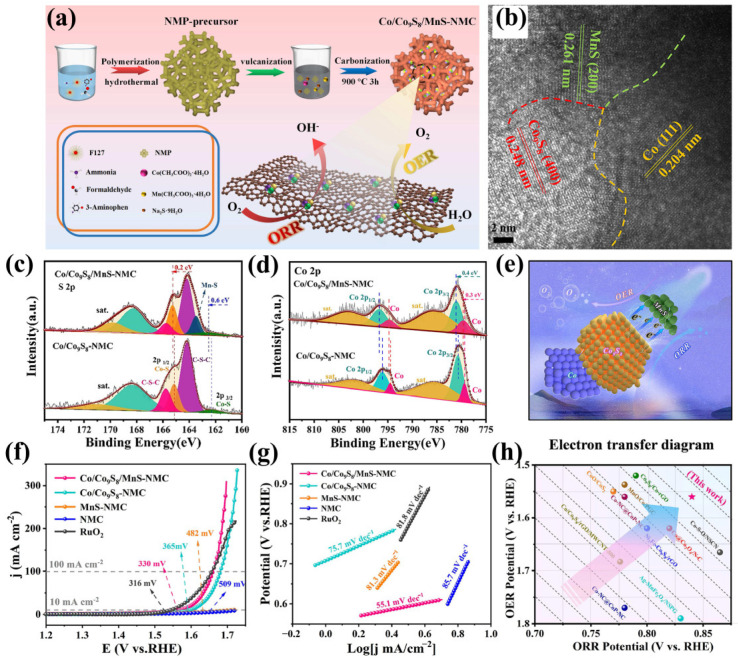
(**a**) Preparation process for Co/Co_9_S_8_/MnS-NMC. (**b**) HRTEM images of the Co/Co_9_S_8_/MnS-NMC. (**c**) S 2p XPS spectra and (**d**) Co 2p XPS spectra for Co/Co_9_S_8_/MnS-NMC and Co/Co_9_S_8_-NMC. (**e**) The electron transfer between Mott–Schottky Co/Co_9_S_8_ structure and MnS species in Co/Co_9_S_8_/MnS-NMC. (**f**) OER polarization curves of Co/Co_9_S_8_/MnS-NMC in 1.0 M KOH, with a scan rate of 10 mV s^−1^. (**g**) Corresponding Tafel plots for all investigated catalysts. (**h**) Comparison of OER and ORR activities of Co/Co_9_S_8_/MnS-NMC with representative samples. Reproduced with permission from Ref. [90]. Copyright 2022, Elsevier B.V.

Banerjee prepared a PbS/MnS heterojunction by chemical sedimentation integration in two steps. The formation of the heterojunction between PbS and MnS can change the conductivity of a single material, adjust the band gap, change the electronic structure of the material, and accelerate the electron transfer rate at the interface between the two components. The surface environment can be changed through electronic interactions to balance the adsorption and desorption strength between the catalyst and the reactant, thus achieving the best electrocatalytic efficiency [86]. In addition, Bo et al. reported a hydrogen evolution rate of 0.0451 mmol g^−1^h^−1^ for a 2D CuInP_2_S_6_ heterostructure [87], while Gan et al. prepared ZnIn_2_S_4_ ultrathin nanosheets that exhibited a hydrogen evolution rate of 3.475 mmol g^−1^h^−1^ [88]. Another example is the H_2_ production rate of 5.92 mmol g^−1^h^−1^ for the CdS/MnS heterostructure prepared by Zhang et al. through the hydrothermal method. The photocarrier can be effectively transferred between CdS and MnS, which is conducive to improve the production performance of photocatalytic H_2_ [89]. The introduction of new metal elements can form an interface between metal materials, which can better transfer electrons and increase the active site. Furthermore, by introducing a MnS phase into Mott–Schottky Co/Co_9_S_8_, Chen et al. designed three-phase Co/Co_9_C_8_/MnS heterojunctions to improve their OER activity. The nitrogen-doped mesoporous polymer (NMP) precursor was first prepared by a hydrothermal method, and then it was vulcanized and carbonized to prepare Co/Co_9_S_8_/MnS-NMC (Figure 8a). It was also observed by HRTEM that MnS was evenly distributed around the Co/Co_9_S_8_ nanostructures, and some obvious interfaces were generated between them, indicating the successful synthesis of the three-phase heterogeneous interface (Figure 8b). Further observation shows that the introduction of MnS in the Co/Co_9_S_8_/MnS-NMC heterostructure can lead to changes in the electronic structure of Co/Co_9_S_8_, as evidenced by the observed positive shift of 0.2 eV in the Co-S bond. Further, it can be concluded that the incorporation of MnS into Mott–Schottky Co/Co_9_S_8_ structures drives electron transfer from the latter to the former at heterogeneous interface channels (Figure 8c–e). When the current density is 10 mA cm^−2^, the OER overpotential is 330 mV and the Tafel slope is 51.3 mV dec^−1^. In summary, incorporating MnS species into the Mott–Schottky Co/Co_9_S_8_ structure enhances electron transfer, accelerates reaction kinetics, and boosts bifunctional ORR/OER activities. (Figure 8f–h) [90]. It can be known from the above examples that the construction of a heterojunction in interface engineering applications can change the original structure of electrons, increase electron transport channels, and thus increase the active sites of catalysts.

### 3.3. Self-Supporting Conductive Substrate Strategy

**Figure 9:**
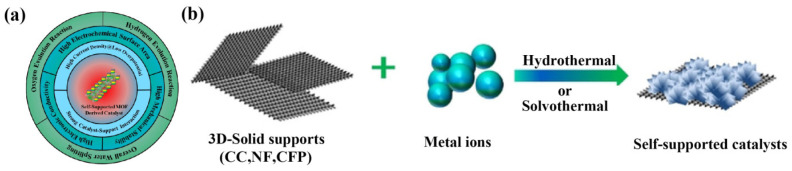
(**a**) Illustration of the interfacial engineering of heterogeneous catalysts. (**b**) Synthesis of self-supported catalyst systems for the electrocatalytic water splitting. Reproduced with permission from Ref. [97]. Copyright 2020, Wiley-VCH.

Most transition metal-based catalysts used for water electrolysis are prepared on binder solid electrode support, which reduces the activity and durability of the catalyst system. In this regard, catalysts derived from a self-loaded metal–organic framework (MOF) have been introduced with a good conductivity, a high electrocatalytic surface area, enhanced mechanical stability, and strong catalyst–support interactions [91,92,93,94,95,96]. MOFs are porous crystalline materials in which the metal centers are connected by coordination bonds via organic bridging ligands to synthesize core–shell and hollow structures. Self-supporting MOF-derived catalyst systems can be designed by controlling surface and interface properties (Figure 9a). A self-supporting catalyst was grown directly on a 3D solid and conductive support in situ without the use of any binders or additives. In general, a catalyst film is grown using nickel foam, carbon cloth, copper foam, and carbon fiber paper as the conductive support (Figure 9b) [97].

**Figure 10:**
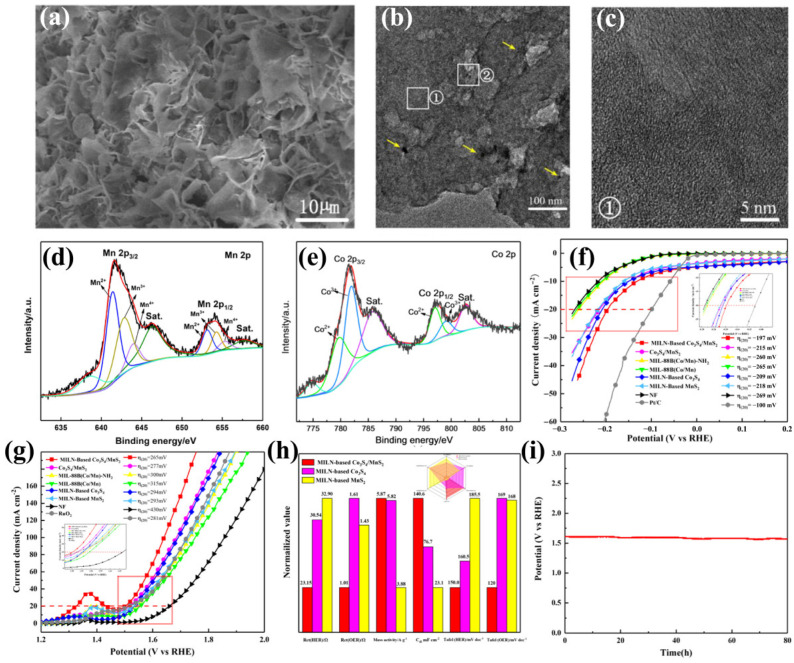
(**a**) FESEM image, (**b**) TEM image, and (**c**) HRTEM of MILN-based Co_3_S_4_/MnS_2_. (**d**,**e**) XPS spectra of Mn 2p, Co 2p. (**f**) HER and (**g**) OER polarization curves in 1.0 M KOH, with a scan rate of 1 mV s^−1^. (**h**) Comparing the activity between MILN-based Co_3_S_4_/MnS_2_, MILN-based Co_3_S_4_ and MILN-based MnS_2_ in 1.0 M KOH. (**i**) Chronopotentiometry test of MILN-based Co_3_S_4_/MnS_2_ at 20 mA cm^−2^ current density under overall water splitting. Reproduced with permission from Ref. [40]. Copyright 2021, Elsevier B.V.

Furthermore, Zhao et al. found that the metal fabrication of a self-supported electrode on the substrate can effectively improve its inherent performance, making the system an efficient electrocatalytic electrode. The increased surface area or roughness and the porous form allow full contact with the electrolyte and the resulting gas to escape, thus improving the catalytic efficiency [96]. Zhang et al. constructed an efficient S-doped carbon bridging semi-crystalline MILN-based Co_3_S_4_/MnS_2_ nanostructure prepared by MIL-88B (Co/Mn)-NH_2_ for integral water electrolysis. Organic ligands can be carbonized to some extent into S-doped carbon. The addition of a self-supporting conductive group accelerates the electron transfer and fully exposes the metal center. Two different transition metal centers with good valence change ability can be found in the composite, and their similar chemical properties enable them to play a synergistic role in the reaction (Figure 10a–e). The formed S-doped carbon can act as a bridge, connecting different metal sulfide nanoparticles and promoting electron transfer. The electrocatalytic performance of Co_3_S_4_/MnS_2_ in the HER can be evaluated by comparing the electrochemical parameters of Co_3_S_4_/MnS_2_ in alkaline solution (1.0 M potassium hydroxide) using a typical three-electrode system. Only 197 mV of overpotential is required at a current density of 20 mA cm^−2^ (Figure 10f,g). It can be found through comparison that the addition of self-supporting conductive structures can improve the catalytic performance, and the interaction between metal centers can play an assisting role (Figure 10h). When the current density is set at 20 mA cm^−2^, the stability test is carried out for 80 h, and the performance can be maintained at 98%. The structure of the material is essentially unchanged, and the stability is excellent (Figure 10i) [40].

Cheng et al. utilized NiMn-based bimetallic-organic framework (NiMn-MOF) nanosheets deposited on multi-channel carbon fibers (MCCF) for electrolytic water splitting. The NiMn-MOF material exhibited a remarkable OER catalytic activity of 280 mV at 10 mA cm^−2^, and DFT calculations revealed that the strong synergistic effect of Ni and Mn bimetals contributed to the formation of crucial *O and *OOH intermediates on the active center. The intimate contact between the highly conductive MCCF substrate and the NiMn-MOF nanosheets facilitated rapid charge transfer and stability, enabling fast OER kinetics [98]. Using MOFs, Li et al. synthesized a 2D ultrathin manganese-doped polyhedron cobalt phosphide (Mn-CoP) with a high specific surface area and abundant catalytic active sites that improved the stability of Mn-CoP [99]. Goswami et al. prepared a self-supported binderless 3D electrode (Mn-MOF/NF) by the hydrothermal method and conducted experiments on complete hydrolysis. Experiments have demonstrated that Mn-MOF materials grown directly on a conductive NF substrate can improve the efficiency of overall water splitting (OWS) by facilitating electron transfer and optimizing electrolyte accessibility. The overpotential of OER was 280 mV at a current density of 20 mA cm^−2^, and the overpotential of the HER was 125 mV at a current density of 10 mA cm^−2^, as well as durability was achieved [100].

### 3.4. Phase Engineering Strategy

Different crystals have varying contributions to the relative catalytic performance of the same material. MnO_x_ and MnS_2_ have different crystal phases and will generate different forms at different temperatures. They have thermodynamically stable crystal phases and many metastable crystal phases. For example, 2D TMDs mainly have the following five crystalline phases: 2H, 3R, 1T, 1T′, and Td. The phase transition of TMDs is a complex process that may involve inter-lattice interactions, interlayer interactions, and interactions between strongly associated electrons. A TMD usually has a lamellar structure and is only one or several atomic layers thick. Two of the most common structures are the 2H and 1T phases that have triangular prismatic and octahedral structural shapes, respectively. The 2H phase can be viewed as an ABA stack, with sulfur molecules in different layers occupying the same position (in-plane position arrangement) and having mirror image symmetry to the metal atomic surface [101,102,103,104,105,106]. The 1T phase is a centrosymmetric structure composed of ABC stacking patterns. In addition to the 2H and 1T phases, another common structure is the 1T′ phase. This structure can be viewed as a periodically distorted 1T phase. For example, converting the TMD of the 2H phase into the TMD of the metal 1T or 1T′ phase can be achieved through phase engineering. Owing to the limited number of active sites and the blocking of ions and mass transport, their electrochemical properties are seriously affected. The phase engineering of nanomaterials (PEN) has become an effective strategy to regulate the properties, functions, and applications of nanomaterials by reasonably adjusting their atomic arrangement [107]. Pujari et al. prepared MnS of different crystalline phases by controlling the temperature, and the materials of different crystalline phases showed different stabilities and catalytic properties. The author confirmed this view and compared the products at different temperatures and found that the overpotential of α-MnS in the steady-state structure was 292 mV [60]. Mori et al. carried out two consecutive phases of displacement transformation from β to α for MnTe with various phase states through the β′ phase; observed its induction process, transition direction, and binding force; and analyzed phase engineering from the ultra-micro perspective [108].

Phase engineering includes direct synthesis and phase transformation. Common methods of phase transformation include direct electron injection (e.g., chemical intercalation, electrochemical intercalation), thermal activation (e.g., annealing treatment, laser irradiation), etc. Zhang et al. investigated the heterogeneous phase structure and phase transition of nanomaterials. In this paper, the latest research progress of PEN is systematically discussed and summarized. Taking representative precious metals and transition metal chalcogenide nanomaterials as examples, various methods of direct synthesis or controlled phase transition to prepare different phase nanomaterials are emphatically discussed, and the properties and applications of different phase nanomaterials are briefly described. The progress in the preparation of amorphous and amorphous-crystalline phase composite heterogeneous junction nanomaterials is also introduced. Finally, combined with the current research status and challenges, the future of PEN research is prospected [109].

## 4. Conclusions and Future Perspectives

In this review, we first introduce the basic mechanism of water electrolysis with manganese-based catalytic materials and then systematically summarize the design strategy of manganese-based electrocatalysts to deal with the existing shortcomings of their applications. Although manganese-based materials have great potential as catalysts for water electrolysis, many challenges remain to be solved [110,111,112]. We concluded that the broad strategy is currently interface engineering and defect engineering. In interface engineering, strong heterogeneous interfaces appear where electrons interact with each other, leading to charge redistribution at their coupling interface, modifying the interface of the material, changing the electron structure, and increasing the rate of electron transfer. As a result, the reaction energy barrier of the rate determining step (RDS) is reduced [113,114,115,116,117,118,119,120]. The addition of MOFs supports the multilayer pore structure, and the mesoporous large pores promote the infiltration of active substances in solution and the mass transport of oxygen, thus accelerating the electrolysis hydrodynamics. The self-supported catalyst directly grows on the 3D solid or conductive support in situ, and the stability problem could be solved without using any binders or additives. Defect engineering can also optimize the charge distribution around the active site, accelerate the charge transfer rate, and change the morphology and structure of the material. The resulting abundant lattice dislocation and surface vacancy are conducive to the acceleration of the electron transfer and enhancement of the catalytic performance of the OER and the HER [66,121,122,123,124,125]. Finally, phase engineering regulates the different morphologies of catalytic materials by changing the reaction temperature. The concept of unconventional phase engineering should be extended to other materials, such as MOFs, covalent organic frameworks, and perovskites. The electrocatalytic performance of the manganese-based materials was significantly improved after the above measures were regulated, and we summarize the electrocatalytic performance of the HER and the OER in Table 1. Given their advantages, constructing these materials will give them unique physical and chemical properties and open up new application prospects and new ways for discovering new functional materials. 

The rapid development of artificial intelligence, advanced material characterization techniques, and complex theoretical calculations will provide tremendous opportunities for researchers to predict new unconventional stages. In the future, interesting electrocatalytic materials will be discovered, achieving a new phase of scalable production with high stability. Although research on the application of manganese-based materials in electrocatalysis has achieved rapid and great progress, some important problems remain to be solved and many challenges to be overcome, such as their relatively wide band gap (e.g., low electron conductivity) and low reactivity due to weak adsorption and bond activation. Further efforts should be made to improve performance. Finally, we propose some questions and possible directions for upgrading manganese-based electrocatalytic materials.

Among the different manganese-based catalysts we reviewed, the actual active sites of the catalysts involved in the reaction are not specifically known through theoretical calculation and observation of the morphology of instruments and equipment. An important part of electrolysis in water is the actual active site of the catalyst: the larger the area involved in the reaction, the more H_2_ and O_2_ are produced. We should not neglect the mechanism of electrocatalytic decomposition of water by manganese-based electrocatalysts. For example, where are the real active sites in the material and are they involved in electrocatalytic reactions? Does the phase of a manganese-based material change during the catalytic reaction? How do electrons migrate across the interface? Therefore, we should introduce other advanced technologies to further study the specific active sites of different catalytic reactions and open up a new world of electrocatalysis.

The greatest problems of manganese transition metal are its poor structural stability, dissolution, and slow ion diffusion. Its catalytic performance and stability can be improved by sulfidation and oxidation when combined with anions, but its stability required by practical engineering applications cannot be reached at present. Manganese-based catalysts are mainly required to have a high stability; although some regulation strategies can provide a certain improvement, the feasibility of practical applications remains to be considered. Through synergistic and localized catalysis, the electrocatalytic activity is improved. This type of catalysis achieves a “win-win” situation by combining high activity and high selectivity. Because metallic manganese is easily oxidized in air, coating can prevent direct contact between active oxide and water molecules. On the basis of the new concept’s transformation path, low water consumption for coal transformation can be realized, providing support for China’s energy revolution.

In conclusion, because manganese-based electrocatalysts have good catalytic hydrolysis ability, researchers have implemented various regulations to improve their performance. Using high-precision calculations and high-throughput screening, we will develop other efficient manganese-based materials as electrocatalysts [126,127,128,129,130,131]. Hence, opportunities and challenges exist in this appealing industry. Furthermore, advanced chemical analysis techniques and standardized test procedures can aid in deepening our understanding of catalytic reactions and designing highly efficient electrocatalytic manganese hydrates to enrich the research of electrolytic water splitting catalysts. Manganese, as a new electrocatalytic material, will be a key technology to solve energy and environmental problems in the future [83,121,132,133,134,135,136,137].

## Figures and Tables

**Table 1:** Summary table of electrocatalytic performance of manganese-based electrocatalysts in HER and OER.

Catalysts	Water Electrolysis Test	η_10_ (mV)	Tafel Slope (mV dec^−1^)	Electrolyte	Ref.
NS-MnO_2_	HER	197	62	1 M KOH	[68]
	OER	320	40		
NF/T(Ni_3_S_2_/MnS-O)	HER	116	54	1 M KOH	[75]
	OER	228	46		
Se-MnS/NiS	HER	56	55	1 M KOH	[76]
	OER	210	50		
MILN-Based Co_3_S_4_/MnS_2_ (20 mA cm^−2^)	HER	197	150	1 M KOH	[40]
	OER	265	120		
Pt/C	HER	46	41	1 M KOH	[40]
RuO_2_	OER	316	82	1 M KOH	[90]
Co/Co_9_S_8_/MnS-NMC	OER	330	51	1 M KOH	[90]
MCCF/NiMn-MOFs	OER	280	86	1 M KOH	[98]
α-MnS	OER	292	70	1 M KOH	[60]

## Data Availability

Not applicable.

## References

[1] Chen P., Hu X. (2020). High-Efficiency Anion Exchange Membrane Water Electrolysis Employing Non-Noble Metal Catalysts. Adv. Energy Mater..

[2] Luo W., Jiang Y., Wang M., Lu D., Sun X., Zhang H. (2023). Design Strategies of Pt-Based Electrocatalysts and Tolerance Strategies in Fuel Cells: A Review. RSC Adv..

[3] Zhao Y., Gao W., Li S., Williams G., Mahadi A., Ma D. (2019). Solar-Versus Thermal-Driven Catalysis for Energy Conversion. Int. J. Hydrog. Energy.

[4] Zhong H., Wang M., Chen G., Dong R., Feng X. (2022). Two-Dimensional Conjugated Metal-Organic Frameworks for Electrocatalysis: Opportunities and Challenges. ACS Nano.

[5] Zhou G., Wang P., Hu B., Shen X., Liu C., Tao W., Huang P., Liu L. (2022). Spin-Related Symmetry Breaking Induced by Half-Disordered Hybridization in Bi_x_Er_2-x_Ru_2_O_7_ Pyrochlores for Acidic Oxygen Evolution. Nat. Commun..

[6] Zhou G., Wang P., Li H., Hu B., Sun Y., Huang R., Liu L. (2021). Spin-State Reconfiguration Induced by Alternating Magnetic Field for Efficient Oxygen Evolution Reaction. Nat. Commun..

[7] Acar C., Dincer I., Naterer G. (2016). Review of Photocatalytic Water-Splitting Methods for Sustainable Hydrogen Production. Int. J. Energy Res..

[8] Anwer H., Park J. (2020). Addressing the OER/HER Imbalance by a Redox Transition-Induced Two-Way Electron Injection in a Bifunctional n–p–n Electrode for Excellent Water Splitting. J. Mater. Chem. A.

[9] Chang G., Zhang H., Yu X. (2022). 2D Metal-Organic Frameworks and Their Derivatives for the Oxygen Evolution Reaction. J. Alloys Compd..

[10] Gao Q., Zhang W., Shi Z., Yang L., Tang Y. (2019). Structural Design and Electronic Modulation of Transition-Metal-Carbide Electrocatalysts toward Efficient Hydrogen Evolution. Adv. Mater..

[11] Lu S., Huynh H., Lou F., Guo K., Yu Z. (2021). Single Transition Metal Atom Embedded Antimonene Monolayers as Efficient Trifunctional Electrocatalysts for the HER, OER and ORR: A Density Functional Theory Study. Nanoscale.

[12] Jiao Y., Zheng Y., Jaroniec M., Qiao S. (2015). Design of Electrocatalysts for Oxygen- and Hydrogen-Involving Energy Conversion Reactions. Chem. Soc. Rev..

[13] Sun T., Mitchell S., Li J., Lyu P., Wu X., Pérez R., Lu J. (2020). Design of Local Atomic Environments in Single-Atom Electrocatalysts for Renewable Energy Conversions. Adv. Mater..

[14] Tian L., Li Z., Xu X., Zhang C. (2021). Advances in Noble Metal (Ru, Rh, and Ir) Doping for Boosting Water Splitting Electrocatalysis. J. Mater. Chem. A.

[15] Bai L., Song A., Wang L., Lei X., Zhang T., Tian H., Liu H., Qin X., Wang G., Shao G. (2022). Enhancement of Hydrogen Desorption for Electrocatalytic Hydrogen Evolution on Nickel-Coupled Graphite Carbon Nitride Catalysts. Ionics.

[16] Bawari S., Kaley N., Pal S., Vineesh T., Ghosh S., Mondal J., Narayanan T. (2018). On the Hydrogen Evolution Reaction Activity of Graphene–hBN Van Der Waals Heterostructures. Phys. Chem. Chem. Phys..

[17] Bi W., Zhang L., Sun Z., Li X., Jin T., Wu X., Zhang Q., Luo Y., Wu C., Xie Y. (2016). Insight into Electrocatalysts as Co-Catalysts in Efficient Photocatalytic Hydrogen Evolution. ACS Catal..

[18] Chen X., Li S., Liu Y., Xie K., Wang Y. (2022). MOF-Derived Mo-CoP@NiFe LDH Hierarchical Nanosheets for High-Performance Hybrid Supercapacitors. J. Alloys Compd..

[19] Guo T., Li L., Wang Z. (2022). Recent Development and Future Perspectives of Amorphous Transition Metal-Based Electrocatalysts for Oxygen Evolution Reaction. Adv. Energy Mater..

[20] Qin Q., Chen L., Wei T., Wang Y., Liu X. (2019). Ni/NiM_2_O_4_ (M = Mn or Fe) Supported on N-Doped Carbon Nanotubes as Trifunctional Electrocatalysts for ORR, OER and HER. Catal. Sci. Technol..

[21] Shang W., Xiao Y., Yu A., Shen H., Cheng Q., Sun Y., Zhang L., Liu L., Li L. (2022). Visible-Light-Enhanced Electrocatalytic Hydrogen Evolution Using Electrodeposited Molybdenum Oxide. J. Electrochem. Soc..

[22] Chen Y., Yang S., Liu H., Zhang W., Cao R. (2021). An Unusual Network of α-MnO_2_ Nanowires with Structure-Induced Hydrophilicity and Conductivity for Improved Electrocatalysis. Chin. J. Catal..

[23] Liu Y., Vijayakumar P., Liu Q., Sakthivel T., Chen F., Dai Z. (2022). Shining Light on Anion-Mixed Nanocatalysts for Efficient Water Electrolysis: Fundamentals, Progress, and Perspectives. Nano-Micro Lett..

[24] Zhao M., Li T., Jia L., Li H., Yuan W., Li C. (2019). Pristine-Graphene-Supported Nitrogen-Doped Carbon Self-Assembled from Glucaminium-Based Ionic Liquids as Metal-Free Catalyst for Oxygen Evolution. ChemSusChem.

[25] Zhao M., Yuan W., Li C. (2017). Controlled Self-Assembly of Ni Foam Supported Poly(Ethyleneimine)/Reduced Graphene Oxide Three-Dimensional Composite Electrodes with Remarkable Synergistic Effects for Efficient Oxygen Evolution. J. Mater. Chem. A.

[26] Yusuf B., Yaseen W., Xie M., Zayyan R., Muhammad A., Nankya R., Xie J., Xu Y. (2023). Recent Advances in Understanding And Design of Efficient Hydrogen Evolution Electrocatalysts for Water Splitting: A Comprehensive Review. Adv. Colloid Interfac..

[27] Xu X., Chen Y., Zhou W., Zhu Z., Su C., Liu M., Shao Z. (2016). A Perovskite Electrocatalyst for Efficient Hydrogen Evolution Reaction. Adv. Mater..

[28] Kong X., Gao Q., Bu S., Xu Z., Shen D., Liu B., Lee C., Zhang W. (2021). Plasma-Assisted Synthesis of Nickel-Cobalt Nitride–Oxide Hybrids for High-Efficiency Electrochemical Hydrogen Evolution. Mater. Today Energy.

[29] Kyriakou V., Garagounis I., Vasileiou E., Vourros A., Stoukides M. (2017). Progress in the Electrochemical Synthesis of Ammonia. Catal. Today.

[30] Wang C., Shang H., Wang Y., Li J., Guo S., Guo J., Du Y. (2021). A General MOF-Intermediated Synthesis of hollow CoFe-Based Trimetallic Phosphides Composed of Ultrathin Nanosheets for Boosting Water Oxidation Electrocatalysis. Nanoscale.

[31] Wang T., Wang T., Chou W., Wu L., Lin S. (2021). First-Principles Investigation of the Hydrogen Evolution Reaction of Transition Metal Phosphides CrP, MnP, FeP, CoP, and NiP. Phys. Chem. Chem. Phys..

[32] Dong C., Ji J., Shen B., Xing M., Zhang J. (2018). Enhancement of H_2_O_2_ Decomposition by the Co-catalytic Effect of WS_2_ on the Fenton Reaction for the Synchronous Reduction of Cr(VI) and Remediation of Phenol. Environ. Sci. Technol..

[33] Lu J., Huang Y., Fu Y., Yan Q., Zeng S. (2021). Synergistic Effect of Photocatalysis and Fenton on Improving the Removal Rate of 4H-SiC during CMP. ECS J. Solid State Sc..

[34] Lkremer M. (2003). The Fenton Reaction. Dependence of the Rate on pH. J. Phys. Chem. A.

[35] Jiang C., Pang S., Ouyang F., Ma J., Jiang J. (2010). A New Insight into Fenton and Fenton-Like Processes for Water Treatment. J. Hazard. Mater..

[36] Wardman P., Candeias L.P. (1996). Fenton Chemistry: An Introduction. Radiat. Res..

[37] Tigwere G., Khan M., Nyamen L., De Souza F., Lin W., Gupta R., Revaprasadu N., Ndifon P. (2023). Transition Metal (Ni, Cu and Fe) Doped MnS Nanostructures: Effect of Doping on Supercapacitance and Water Splitting. Mater. Sci. Semicond. Process..

[38] Xu J., Wang Y., Song N., Luo S., Xu B., Zhang J., Wang F. (2022). Doping of the Mn Vacancy of Mn_2_B_2_ with a Single Different Transition Metal Atom as the Dual-Function Electrocatalyst. Phys. Chem. Chem. Phys..

[39] Zhang W., Zong L., Fan K., Cui L., Zhang Q., Zhao J., Wang L., Feng S. (2021). Enabling Highly Efficient Electrocatalytic Oxygen Reduction and Evolution Reaction by Established Strong MnO/Co-Support Interaction. J. Alloys Compd..

[40] Zhang R., Yu Z., Jiang R., Huang J., Hou Y., Yang F., Zhu H., Zhang B., Huang Y., Ye B. (2021). Dual Synergistic Effect of S-Doped Carbon Bridged Semi Crystalline MILN-Based Co_3_S_4_/MnS_2_ Nanostructure in Electrocatalytic Overall Water Splitting. Electrochim. Acta.

[41] Wang X., Huang G., Pan Z., Kang S., Ma S., Shen P., Zhu J. (2022). One-Pot Synthesis of Mn_2_P-Mn_2_O_3_ Heterogeneous Nanoparticles in a P, N -Doped Three-Dimensional Porous Carbon Framework as a Highly Efficient Bifunctional Electrocatalyst for Overall Water Splitting. Chem. Eng. J..

[42] Chen L., Ren S., Xing X., Yang J., Li J., Yang J., Liu Q. (2022). Effect of MnO_2_ Crystal Types on CeO_2_@MnO_2_ Oxides Catalysts for Low-Temperature NH_3_-SCR. J. Environ. Chem. Eng..

[43] Hatakeyama T., Okamoto N., Ichitsubo T. (2022). Thermal Stability of MnO_2_ Polymorphs. J. Solid State Chem..

[44] Lei N., Qiao Y., Liu G., Xu R., Jiang G., Demir M., Ma P. (2022). MnO_2_ Modified Perovskite Oxide SrCo_0.875_Nb_0.125_O_3_ as Supercapacitor Electrode Material. Mater. Chem. Phys..

[45] Wang J., Yang H., Zhou C., Xu J., Wang J., Ren Y. (2020). Template-Assisted Preparation of MnO_2_@MnO_2_ Hollow Nanospheres and Their Research of Capacitance Performance. Mater. Lett..

[46] Yang R., Fan Y., Ye R., Tang Y., Cao X., Yin Z., Zeng Z. (2021). MnO_2_-Based Materials for Environmental Applications. Adv. Mater..

[47] Leong Z., Yang H. (2019). A Study of MnO_2_ with Different Crystalline Forms for Pseudocapacitive Desalination. ACS Appl. Mater. Interfaces.

[48] Li Y., Zhu S., Liu Z. (2016). Reaction Network of Layer-to-Tunnel Transition of MnO_2_. J. Am. Chem. Soc..

[49] Sari F., So P., Ting J. (2017). MnO_2_ with Controlled Phase for Use in Supercapacitors. J. Am. Ceram. Soc..

[50] Yang Y., Chuan X. (2017). Study on Electrochemical Properties of MnO_2_/MoS_2_ Composites. Acta Geol. Sin.—Engl. Ed..

[51] Yuan Y., He K., Byles B., Liu C., Amine K., Lu J., Pomerantseva E., Shahbazian-Yassar R. (2019). Deciphering the Atomic Patterns Leading to MnO_2_ Polymorphism. Chem.

[52] Cheng F., Su Y., Liang J., Tao Z., Chen J. (2009). MnO_2_-Based Nanostructures as Catalysts for Electrochemical Oxygen Reduction in Alkaline Media. Chem. Mater..

[53] Chen K., Dong Noh Y., Li K., Komarneni S., Xue D. (2013). Microwave–Hydrothermal Crystallization of Polymorphic MnO_2_ for Electrochemical Energy Storage. J. Phys. Chem. C.

[54] Barhoumi M., Lazaar K., Said M. (2018). Electronic and Vibrational Properties of TMDs Heterogeneous Bilayers, Nontwisted Bilayers Silicene/TMDs Heterostructures and Photovoltaic Heterojunctions of Fullerenes with TMDs Monolayers. Phys. E Low-Dimens. Syst. Nanostructures.

[55] Guan J., Huang C., Deng K., Kan E. (2019). First-Principles Prediction of Room-Temperature Ferromagnetic Semiconductor MnS_2_ via Isovalent Alloying. J. Phys. Chem. C.

[56] Rana A., Jeong M., Noh Y., Park H., Baik J., Choi K. (2022). Phase-Tuned MoS_2_ and Its Hybridization with Perovskite Oxide as Bifunctional Catalyst: A Rationale for Highly Stable and Efficient Water Splitting. ACS Appl. Mater. Interfaces.

[57] Chen D., Wang C., Liu F., Peng C. (2023). Variation of Magnetism in a Two-Dimensional Non-Van Der Waals MnS_2_ Bilayer. Appl. Surf. Sci..

[58] Chen D., Wang C., Li J., Liu F. (2022). Variation of Magnetism in Two-Dimensional MnS_2_ Thin Films. J. Magn. Magn. Mater..

[59] Durkee D., Smith D., Torchio R., Petitgirard S., Briggs R., Kantor I., Evans S., Chatterji T., Irifune T., Pascarelli S. (2019). Electronic Origins of the Giant Volume Collapse in the Pyrite Mineral MnS_2_. J. Solid State Chem..

[60] Pujari R., Gund G., Patil S., Park H., Lee D. (2020). Anion-Exchange Phase Control of Manganese Sulfide for Oxygen Evolution Reaction. J. Mater. Chem. A.

[61] Sun M., Gao R., Liu X., Gao R., Wang L. (2020). Manganese-Based Oxygen Evolution Catalysts Boosting Stable Solar-Driven Water Splitting: MnSe as an Intermetallic Phase. J. Mater. Chem. A.

[62] Tian L., Zhai X., Wang X., Li J., Li Z. (2020). Advances in Manganese-Based Oxides for Oxygen Evolution Reaction. J. Mater. Chem. A.

[63] Wang P., Zhang S., Wang Z., Mo Y., Luo X., Yang F., Lv M., Li Z., Liu X. (2023). Manganese-Based Oxide Electrocatalysts for the Oxygen Evolution Reaction: A Review. J. Mater. Chem. A.

[64] Kumar A., Raizada P., Khan A., Nguyen V., Van Le Q., Singh A., Saini V., Selvasembian R., Huynh T., Singh P. (2021). Phenolic Compounds Degradation: Insight into the role and Evidence of Oxygen Vacancy Defects Engineering on Nanomaterials. Sci. Total Environ..

[65] Rudolph P. (2016). Fundamentals and Engineering of Defects. Prog. Cryst. Growth Charact. Mater..

[66] Yan D., Xia C., Zhang W., Hu Q., He C., Xia B., Wang S. (2022). Cation Defect Engineering of Transition Metal Electrocatalysts for Oxygen Evolution Reaction. Adv. Energy Mater..

[67] Zhang T., Wu S., Li N., Chen G., Hou L. (2023). Applications of Vacancy Defect Engineering in Persulfate Activation: Performance and Internal Mechanism. J. Hazard. Mater..

[68] Zhao Y., Chang C., Teng F., Zhao Y., Chen G., Shi R., Waterhouse G., Huang W., Zhang T. (2017). Defect-Engineered Ultrathin δ-MnO_2_Nanosheet Arrays as Bifunctional Electrodes for Efficient Overall Water Splitting. Adv. Energy Mater..

[69] Gupta P., Bhandari A., Saha S., Bhattacharya J., Pala R. (2019). Modulating Oxygen Evolution Reactivity in MnO_2_ through Polymorphic Engineering. J. Phys. Chem. C.

[70] Mo S., Zhang Q., Li J., Sun Y., Ren Q., Zou S., Zhang Q., Lu J., Fu M., Mo D. (2020). Highly Efficient Mesoporous MnO_2_ Catalysts for the Total Toluene Oxidation: Oxygen-Vacancy Defect Engineering and Involved Intermediates Using in Situ Drifts. Appl. Catal. B.

[71] Kölbach M., Fiechter S., Van De Krol R., Bogdanoff P. (2017). Evaluation of Electrodeposited α-Mn_2_O_3_ as a Catalyst for the Oxygen Evolution Reaction. Catal. Today.

[72] Mattelaer F., Bosserez T., Rongé J., Martens J., Dendooven J., Detavernier C. (2016). Manganese Oxide Films with Controlled Oxidation State for Water Splitting Devices through a Combination of Atomic Layer Deposition and Post-Deposition Annealing. RSC Adv..

[73] Luo X., Wang J., Liang Z., Chen S., Liu Z., Xu C. (2017). Manganese Oxide with Different Morphology as Efficient Electrocatalyst for Oxygen Evolution Reaction. Int. J. Hydrog. Energy.

[74] Ye Z., Li T., Ma G., Dong Y., Zhou X. (2017). Metal-Ion (Fe, V, Co, and Ni)-Doped MnO_2_ Ultrathin Nanosheets Supported on Carbon Fiber Paper for the Oxygen Evolution Reaction. Adv. Funct. Mater..

[75] Zhang Y., Fu J., Zhao H., Jiang R., Tian F., Zhang R. (2019). Tremella-Like Ni_3_S_2_/MnS with Ultrathin Nanosheets and Abundant Oxygen Vacancies Directly Used for High Speed Overall Water Splitting. Appl. Catal. B.

[76] Zhu J., Sun M., Liu S., Liu X., Hu K., Wang L. (2019). Study of Active Sites on Se-MnS/NiS Heterojunctions as Highly Efficient Bifunctional Electrocatalysts for Overall Water Splitting. J. Mater. Chem. A.

[77] Zhao M., Zhang S., Lin J., Hu W., Li C. (2022). Synergic Effect of Fe-Doping And Ni_3_S_2_/MnS Heterointerface to Boost Efficient Oxygen Evolution Reaction. Electrochim. Acta.

[78] Gupta S., Zhao S., Wang X., Hwang S., Karakalos S., Devaguptapu S., Mukherjee S., Su D., Xu H., Wu G. (2017). Quaternary FeCoNiMn-Based Nanocarbon Electrocatalysts for Bifunctional Oxygen Reduction and Evolution: Promotional Role of Mn Doping in Stabilizing Carbon. ACS Catal..

[79] Du Y., Li B., Xu G., Wang L. (2022). Recent Advances in Interface Engineering Strategy for Highly-Efficient Electrocatalytic Water Splitting. InfoMat.

[80] Jung J., Chang M., Yoon H. (2018). Interface Engineering Strategies for Fabricating Nanocrystal-Based Organic–Inorganic Nanocomposites. Appl. Sci..

[81] Mao X., Shen P. (2022). Interface Engineering of NiMoS_x_ Heterostructure Nanorods for Efficient Oxygen Evolution Reaction. J. Colloid Interface Sci..

[82] Pan J., Wang P., Chen Z., Yu Q., Wang P., Zhu M., Zhao W., Wang J., Zheng Y., Li C. (2021). The Pt-free 1T/2H-MoS_2_/CdS/MnO_x_ Hollow Core-Shell Nanocomposites Toward Overall Water Splitting via HER/OER Synergy of 1T-MoS_2_/MnO_x_. Mater. Today Chem..

[83] Shao Q., Wang P., Huang X. (2019). Opportunities and Challenges of Interface Engineering in Bimetallic Nanostructure for Enhanced Electrocatalysis. Adv. Funct. Mater..

[84] Wang C., Bai S., Xiong Y. (2015). Recent Advances in Surface And Interface Engineering for Electrocatalysis. Chin. J. Catal..

[85] Zhang Y., Lin Y., Duan T., Song L. (2021). Interfacial Engineering of Heterogeneous Catalysts for Electrocatalysis. Mater. Today.

[86] Banerjee A. (2018). Electrical and Optoelectronic Properties of Chemically Prepared PbS/MnS Heterojunction. J. Electron. Mater..

[87] Lin B., Chaturvedi A., Di J., You L., Lai C., Duan R., Zhou J., Xu B., Chen Z., Song P. (2020). Ferroelectric-Field Accelerated Charge Transfer in 2D CuInP_2_S_6_ Heterostructure for Enhanced Photocatalytic H_2_ Evolution. Nano Energy.

[88] Zuo G., Wang Y., Teo W., Xie A., Guo Y., Dai Y., Zhou W., Jana D., Xian Q., Dong W. (2020). Ultrathin ZnIn_2_S_4_ Nanosheets Anchored on Ti_3_C_2_T_X_ MXene for Photocatalytic H_2_ Evolution. Angew. Chem. Int. Ed. Engl..

[89] Zhang H., Feng Q., Zhang Y., Zhang J., Wu X., Li Y., Yin L., Huang J., Kong X. (2022). A CdS/MnS p–n Heterojunction with a Directional Carrier Diffusion Path for Efficient Photocatalytic H_2_ Production. Inorg. Chem. Front..

[90] Chen K., Wang X., Zhang C., Xu R., Wang H., Chu L., Huang M. (2022). Three-Phases Co/Co_9_S_8_/MnS Heterostructures Engineering for Boosted ORR/OER Activities in Zn–Air Batteries. Mater. Today Energy.

[91] Lin C., He X., Li H., Zou J., Que M., Tian J., Qian Y. (2021). Tunable Metal–Organic Framework Nanoarrays on Carbon Cloth Constructed by A Rational Self-Sacrificing Template for Efficient and Robust Oxygen Evolution Reactions. CrystEngComm.

[92] Liu P., Jing P., Xu X., Liu B., Zhang J. (2021). Structural Reconstruction of Ce-MOF with Active Sites for Efficient Electrocatalytic N_2_ Reduction. ACS Appl. Energy Mater..

[93] Wang L., Wang A., Xue Z., Hu J., Han S., Wang G. (2022). Ultrathin Two-Dimensional Polyoxometalate-Based Metal–Organic Framework Nanosheets for Efficient Electrocatalytic Hydrogen Evolution. Inorg. Chem..

[94] Wang X., Han Y., Zhang J., Li Z., Li T., Zhao X., Liu W. (2019). Influence of Electropolished Copper Substrate on Morphology of Electroplating Self-Supporting Ni Films. Nucl. Instrum. Meth. A.

[95] Xu R., Wu R., Shi Y., Zhang J., Zhang B. (2016). Ni_3_Se_2_ Nanoforest/Ni Foam as a Hydrophilic, Metallic, and Self-Supported Bifunctional Electrocatalyst for Both H_2_ and O_2_ Generations. Nano Energy.

[96] Zhao Y., Wei S., Pan K., Dong Z., Zhang B., Wu H., Zhang Q., Lin J., Pang H. (2021). Self-Supporting Transition Metal Chalcogenides on Metal Substrates for Catalytic Water Splitting. Chem. Eng. J..

[97] Singh B., Indra A. (2020). Designing Self-Supported Metal-Organic Framework Derived Catalysts for Electrochemical Water Splitting. Chem. Asian J..

[98] Cheng W., Lu X., Luan D., Lou X. (2020). NiMn-Based Bimetal-Organic Framework Nanosheets Supported on Multi-Channel Carbon Fibers for Efficient Oxygen Electrocatalysis. Angew. Chem. Int. Ed..

[99] Li Y., Jia B., Chen B., Liu Q., Cai M., Xue Z., Fan Y., Wang H., Su C., Li G. (2018). MOF-Derived Mn Doped Porous Cop Nanosheets as Efficient and Stable Bifunctional Electrocatalysts for Water Splitting. Dalton Trans..

[100] Goswami A., Ghosh D., Pradhan D., Biradha K. (2022). In Situ Grown Mn(II) MOF upon Nickel Foam Acts as a Robust Self-Supporting Bifunctional Electrode for Overall Water Splitting: A Bimetallic Synergistic Collaboration Strategy. ACS Appl. Mater. Interfaces.

[101] Chen H., Zhang M., Wang Y., Sun K., Wang L., Xie Z., Shen Y., Han X., Yang L., Zou X. (2022). Crystal Phase Engineering of Electrocatalysts for Energy Conversions. Nano Res..

[102] Chen Y., Chen J., Liu J., Lin Z., Hu X., Lin X., Xu Z., Zeb A. (2022). Metal-Organic Framework-Derived Mixed-Phase Anatase/Rutile TiO_2_ towards Boosted Lithium Storage: Surface Engineering and Design Strategy Through Crystal Phase Transition. Mater. Today Nano.

[103] Chu X., Wang L., Li J., Xu H. (2023). Strategies for Promoting Catalytic Performance of Ru-based Electrocatalysts towards Oxygen/Hydrogen Evolution Reaction. Chem. Rec..

[104] Lee M., Yang J., Kwon H., Jang H. (2022). Crystal Facet and Phase Engineering for Advanced Water Splitting. CrystEngComm.

[105] Li M., Shu C., Hu A., Li J., Liang R., Long J. (2020). Invigorating the Catalytic Activity of Cobalt Selenide via Structural Phase Transition Engineering for Lithium–Oxygen Batteries. ACS Sustain. Chem. Eng..

[106] Zhang Q., Zhang M., Chen T., Li L., Shi S., Jiang R. (2022). Unconventional Phase Engineering of Fuel-Cell Electrocatalysts. J. Electroanal. Chem..

[107] Zhang M., Chen Y., Yang D., Li J. (2020). High Performance MnO_2_ Supercapacitor Material Prepared by Modified Electrodeposition Method with Different Electrodeposition Voltages. J. Energy Storage.

[108] Mori S., Ando D., Sutou Y. (2020). Sequential Two-Stage Displacive Transformation from β to α via β′Phase in Polymorphic MnTe Film. Mater. Des..

[109] Chen Y., Lai Z., Zhang X., Fan Z., He Q., Tan C., Zhang H. (2020). Phase Engineering of Nanomaterials. Nat. Rev. Chem..

[110] Bacirhonde P., Dzade N., Chalony C., Park J., Jeong E., Afranie E., Lee S., Kim C., Kim D., Park C. (2022). Reduction of Transition-Metal Columbite-Tantalite as a Highly Efficient Electrocatalyst for Water Splitting. ACS Appl. Mater. Interfaces.

[111] Luo J., Guo W., Zhang Q., Wang X., Shen L., Fu H., Wu L., Chen X., Luo H., Li N. (2020). One-Pot Synthesis of Mn–Fe Bimetallic Oxide Heterostructures as Bifunctional Electrodes for Efficient Overall Water Splitting. Nanoscale.

[112] Shen L., Zhang Q., Luo J., Fu H., Chen X., Wu L., Luo H., Li N. (2021). Heteroatoms Adjusting Amorphous FeMn-Based Nanosheets via a Facile Electrodeposition Method for Full Water Splitting. ACS Sustain. Chem. Eng..

[113] Chen Y., Cai Z., Wang D., Yan Y., Wang P., Wang X. (2021). Air-Stable Mn doped CuCl/CuO Hybrid Triquetrous Nanoarrays as Bifunctional Electrocatalysts for Overall Water Splitting. Chem. Asian J..

[114] Hayat A., Sohail M., Ali H., Taha T., Qazi H., Ur Rahman N., Ajmal Z., Kalam A., Al-Sehemi A., Wageh S. (2022). Recent Advances and Future Perspectives of Metal-Based Electrocatalysts for Overall Electrochemical Water Splitting. Chem. Rec..

[115] Li Y., Li R., Wang D., Xu H., Meng F., Dong D., Jiang J., Zhang J., An M., Yang P. (2021). A Review: Target-Oriented Transition Metal Phosphide Design and Synthesis for Water Splitting. Int. J. Hydrog. Energy.

[116] Paul A., Symes M. (2021). Decoupled Electrolysis for Water Splitting. Curr. Opin. Green Sustain. Chem..

[117] Rosman N., Yunus R., Shah N., Shah R., Arifin K., Minggu L., Ludin N. (2022). An Overview of Co-Catalysts on Metal Oxides for Photocatalytic Water Splitting. Int. J. Energy Res..

[118] Tang T., Jiang W., Niu S., Liu N., Luo H., Chen Y., Jin S., Gao F., Wan L., Hu J. (2017). Electronic and Morphological Dual Modulation of Cobalt Carbonate Hydroxides by Mn Doping toward Highly Efficient and Stable Bifunctional Electrocatalysts for Overall Water Splitting. J. Am. Chem. Soc..

[119] Xiao K., Wei J., Han W., Liu Z. (2021). Bimetallic Sulfide Interfaces: Promoting Destabilization of Water Molecules for Overall Water Splitting. J. Power Sources.

[120] Zhang H., Li H., Zhou Y., Tan F., Dai R., Liu X., Hu G., Jiang L., Chen A., Wu R. (2023). Heterostructured Bimetallic Phosphide Nanowire Arrays with Lattice-Torsion Interfaces for Efficient Overall Water Splitting. J. Energy Chem..

[121] Bai S., Zhang N., Gao C., Xiong Y. (2018). Defect Engineering in Photocatalytic Materials. Nano Energy.

[122] Hasan M., Gomaa A., Khedr G., Salem K., Shaheen B., Allam N. (2022). Highly Durable Compositionally Variant Bifunctional Tetrametallic Ni–Co–Mn–Fe Phosphide Electrocatalysts Synthesized by a Facile Electrodeposition Method for High-Performance Overall Water Splitting. Energy Fuels.

[123] Huang H., Hu X., Hou Z., Yang D., Xiang D., Hu L. (2022). Interfacial Construction and Lattice Distortion-Triggered Bifunctionality of Mn-NiS/Mn-Ni_3_S_4_ for H_2_ Production. Fuel.

[124] Kotha V., Karajagi I., Ghosh P., Panchakarla L. (2022). Potassium-Substituted LaMnO_3_ as a Highly Active and Exceptionally Stable Electrocatalyst toward Bifunctional Oxygen Reduction and Oxygen Evolution Reactions. ACS Appl. Energy Mater..

[125] Liu F., Shi C., Guo X., He Z., Pan L., Huang Z., Zhang X., Zou J. (2022). Rational Design of Better Hydrogen Evolution Electrocatalysts for Water Splitting: A Review. Adv. Sci..

[126] Adegoke K., Maxakato N. (2022). Empirical Analysis and Recent Advances in Metal-Organic Framework-Derived Electrocatalysts for Oxygen Reduction, Hydrogen and Oxygen Evolution Reactions. Mater. Chem. Phys..

[127] Du Y., Zhang M., Wang Z., Liu Y., Liu Y., Geng Y., Wang L. (2019). A Self-Templating Method for Metal–Organic Frameworks to Construct Multi-Shelled Bimetallic Phosphide Hollow Microspheres as Highly Efficient Electrocatalysts for Hydrogen Evolution Reaction. J. Mater. Chem. A.

[128] Li Z., Hu M., Wang P., Liu J., Yao J., Li C. (2021). Heterojunction Catalyst in Electrocatalytic Water Splitting. Coord. Chem. Rev..

[129] Liu W., Cao D., Cheng D. (2021). Review on Synthesis and Catalytic Coupling Mechanism of Highly Active Electrocatalysts for Water Splitting. Energy Technol..

[130] Qin D., Tang Y., Ma G., Qin L., Tao C., Zhang X., Tang Z. (2021). Molecular Metal Nanoclusters for ORR, HER and OER: Achievements, Opportunities and Challenges. Int. J. Hydrog. Energy.

[131] Wang X., Li F., Li W., Gao W., Tang Y., Li R. (2017). Hollow Bimetallic Cobalt-Based Selenide Polyhedrons Derived from Metal–Organic Framework: An Efficient Bifunctional Electrocatalyst for Overall Water Splitting. J. Mater. Chem. A.

[132] Cao L., Zhang B., Zhao S. (2022). Cation-Tuning Engineering on Metal Oxides for Oxygen Electrocatalysis. Chem. Eur. J..

[133] Chandrasekaran S., Hu R., Yao L., Sui L., Liu Y., Abdelkader A., Li Y., Ren X., Deng L. (2023). Mutual Self-Regulation of d-Electrons of Single Atoms and Adjacent Nanoparticles for Bifunctional Oxygen Electrocatalysis and Rechargeable Zinc-Air Batteries. Nano-Micro Lett..

[134] Huo X., Yu H., Xing B., Zuo X., Zhang N. (2022). Review of High Entropy Alloys Electrocatalysts for Hydrogen Evolution, Oxygen Evolution, and Oxygen Reduction Reaction. Chem. Rec..

[135] Wang J., Fan Y., Qi S., Li W., Zhao M. (2020). Bifunctional HER/OER or OER/ORR Catalytic Activity of Two-Dimensional TM_3_(HITP)_2_ with TM = Fe-Zn. J. Phys. Chem. C.

[136] Xu Y., Zhang X., Liu Y., Wang R., Yang Y., Chen J. (2022). A Critical Review of Research Progress for Metal Alloy Materials in Hydrogen Evolution and Oxygen Evolution Reaction. Environ. Sci. Pollut. Res..

[137] Zhang N., Jiang R. (2021). Interfacial Engineering of Metal/Metal Oxide Heterojunctions toward Oxygen Reduction and Evolution Reactions. ChemPlusChem.

